# Supplementary material for: Integrating multimodal data through interpretable heterogeneous ensembles
Source: Bioinform Adv. 2022 Sep 12;2(1):vbac065. doi: 10.1093/bioadv/vbac065 (PMC9495448; doi:10.1093/bioadv/vbac065)
Supplement: vbac065_Supplementary_Data [file vbac065_supplementary_data.pdf]

Supplementary Materials

Compliance with DOME recommendations

Our study followed the Data, Optimization, Model and Evaluation (DOME) recommendations (Walsh *et al.* (2021)), as detailed below:

- **Data**: The evaluation of EI for protein function prediction (PFP) is based on publicly available STRING network and Gene Ontology annotation data, both described in Section 2.3.1. The same section also describes the number of proteins and features covered by the STRING data, as well as the distribution of proteins across the GO terms. We have shared all the data used in the PFP experiments with the public EI GitHub repository (https://github.com/GauravPandeyLab/ensemble_integration).

  The electronic health record (EHR) and outcome data used in the COVID-19 mortality prediction experiments were obtained from the Mount Sinai Data Warehouse, and were prepared by expert clinicians and informaticians. These data, including the distribution of patients over the values of the mortality outcome (alive and deceased) are decribed in Section 2.3.2. However, due to restrictions to protect patient privacy, we are unable to publicly share these data.
- **Optimization**: The algorithms used for building the local and EI ensemble models in this study are listed in Section 2.1. The default parameters of these algorithms in the respective public libraries they were adopted from (Weka and scikit-learn respectively) were used. The only exceptions were specifying C=0.001 for SVM and M=100 for LR to control time to convergence, based on our previous experience with these algorithms. However, we did not optimize the parameters of any of the prediction algorithms used for each dataset and/or label individually to avoid overfitting.

  All the training of the local and EI ensemble models was conducted in a nested cross-validation (Nested CV; Section 2.4) setup. In this setup, the whole dataset is split into five outer folds, which are further divided into inner folds. The inner folds are used for training the local models, while the outer folds are used for training and evaluating the ensembles. Nested CV also helps reduce overfitting during heterogeneous ensemble learning by separating the set of examples on which the local and ensemble models are trained and evaluated (Whalen *et al.* (2016)).

  All the algorithms and their parameters are included in the EI code provided at the public GitHub repository mentioned above. Users of the code are also able to change these settings as they desire.
- **Model**: Note that our study was focused on proposing and evaluating prediction algorithms, such as EI and benchmarks like deepNF and Mashup, and not to propose one or more specific models for our target problems. The only exception to this was the EI-based COVID-19 mortality prediction model that was interpreted in Section 3.3. We have shared this model through the GitHub repository mentioned above. We also hope that the results of the interpretation of this model will help shed light on COVID-19 pathophysiology, as well as help other researchers design and conduct related studies. More importantly, we hope that our EI framework provides a novel, reliable methodology for building specific models in other studies.
- **Evaluation**: As explained in Section 2.4, as well as relevant subsections of Section 3 (Results), we rigorously evaluated our proposed EI framework, and compared them with relevant benchmark approaches. Specifically, we used the Nested CV setup described above to fairly evaluation all the algorithms, as well as reduce overfitting in the process. We also used a variety of evaluation metrics, most prominently F_{max}, which was recommended by the Critical Assessment of Protein Function Annotation (CAFA) exercise (Radivojac *et al.* (2013)) for the evaluation of supervised methods for unbalanced classes, like in PFP. We also evaluated the consistency of our EI interpretation method with other methods and evidence in the literature (Section 3.3). Thus, consistent with the focus of our study, we rigorously evaluated all the algorithms tested, and assessed the results they generated.

We hope that the substantial details we have provided in accordance with the DOME recommendations for our study will aid its reproducibility and utility.

Supplementary Figures

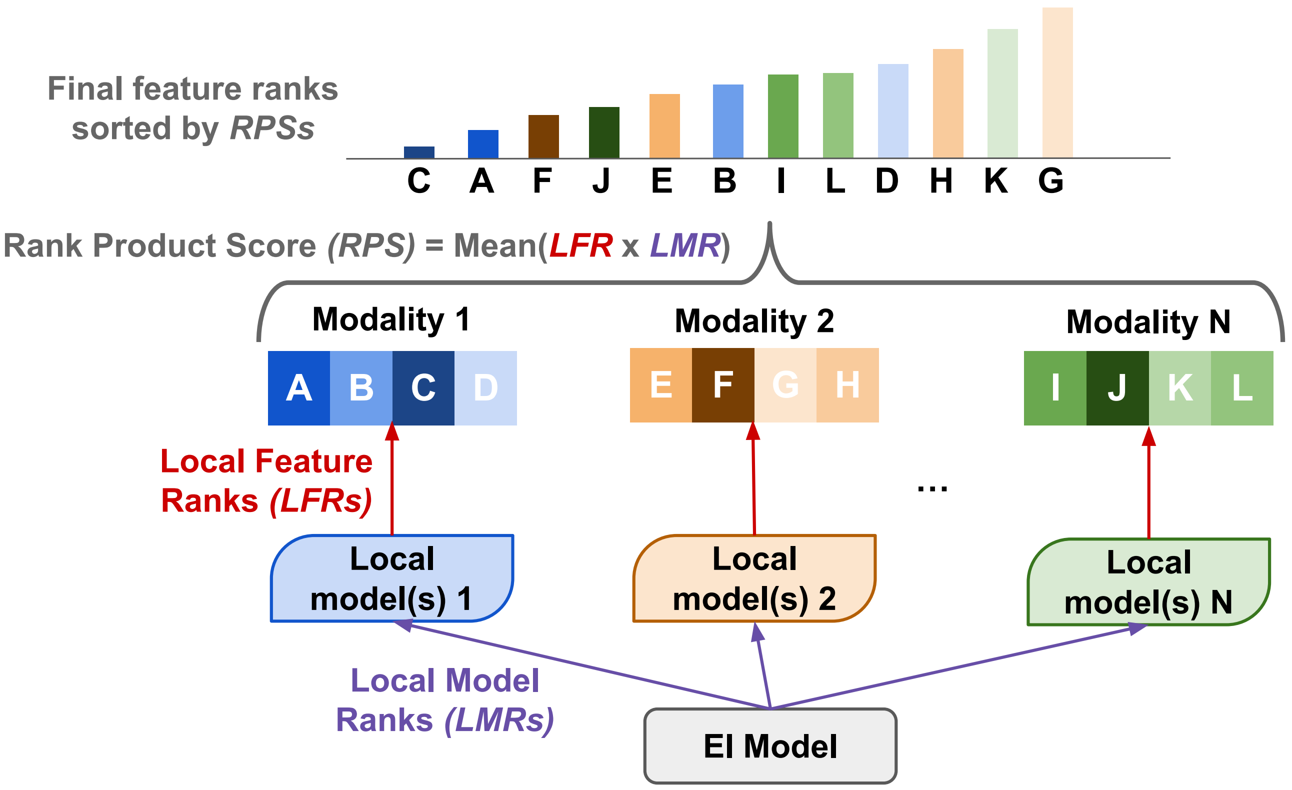

Supplementary Fig. 1: **Overview of the EI model interpretation method.** The method is based on local model (LMRs, purple arrow) and feature (LFRs, red arrow) ranks. LMR denotes the importance of a local model derived from one of the data modality (e.g., Local model(s) 1 derived from Modality 1) to the final EI model, while LFR denotes the contribution of each feature in the corresponding data modality (e.g., A-D in Modality 1) to a local model. The method averages the product of the LMR and LFR for each valid pair of local model and feature into a rank product score (RPS). The final ranking of all the features in terms of their importance is determined by sorting the RPSs in ascending order.

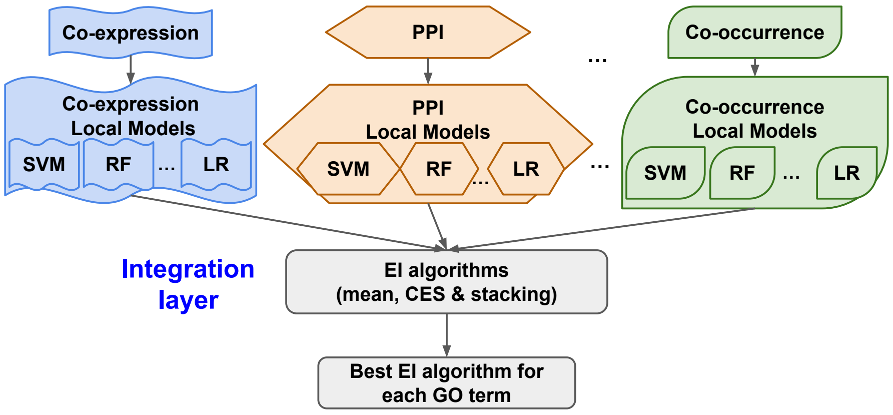

(a) Identifying the best-performing EI algorithm.

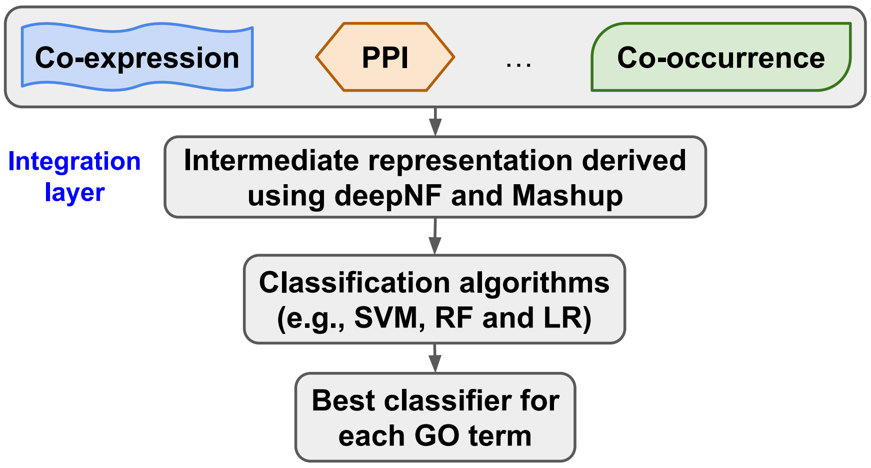

(b) Identifying the best-performing classification algorithm for the integrated networks (intermediate representations) derived using deepNF and Mashup.

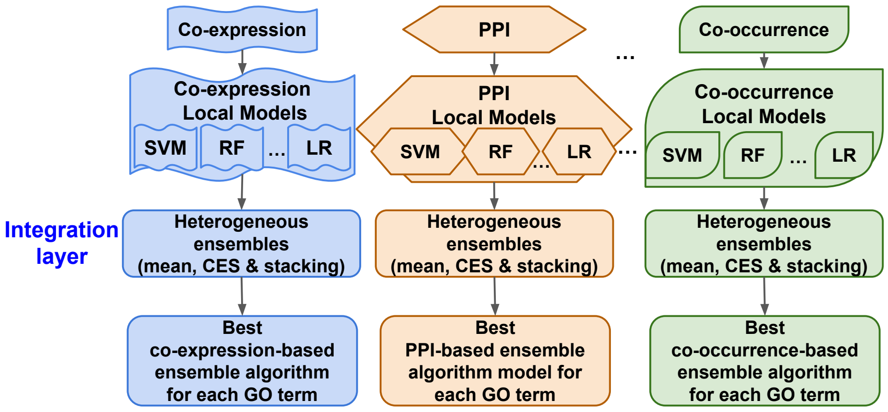

(c) Identifying the best-performing heterogeneous ensemble algorithms for the individual data modalities in STRING.

Supplementary Fig. 2: **Overview of the workflow for identifying the best-performing algorithms for protein function prediction.** These algorithms, namely (a) EI, (b) classifiers on integrated networks derived using deepNF and Mashup and (c) heterogeneous ensembles applied to the individual data modalities were applied to the STRING data as described in Section 2.3.1. Also marked in the workflows are the layers (steps) at which data and/or information were integrated. Based on the cross-validation results obtained, we identified and compared the best-performing algorithms in each of these categories for each GO term.

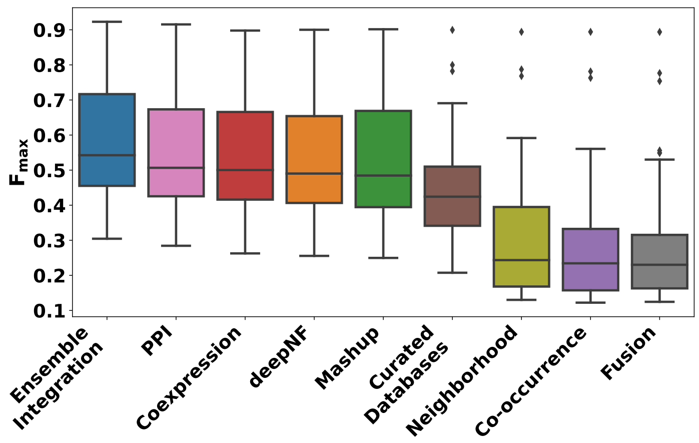

(a) GO terms with more than 1000 annotations (FDR of EI vs deepNF $= 9.05 \times 10^{-14}$, EI vs Mashup $< 2 \times 10^{-16}$, EI vs all individual modalities $< 3.19 \times 10^{-6}$).

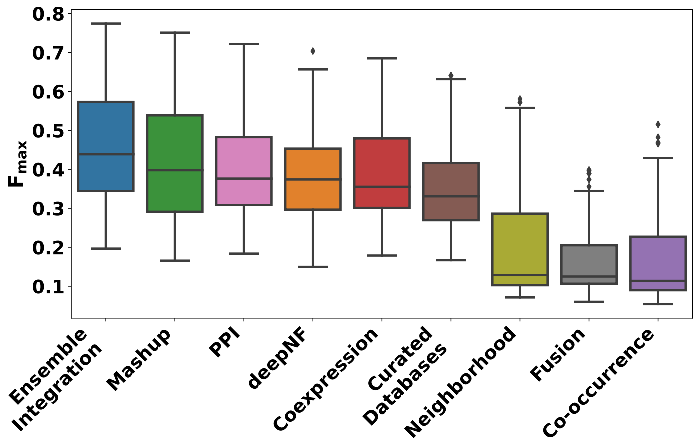

(b) GO terms with 500 to 1000 annotations (FDR of EI vs deepNF $= 8.86 \times 10^{-14}$, EI vs Mashup $= 1.09 \times 10^{-13}$, EI vs all individual modalities $< 8.77 \times 10^{-12}$).

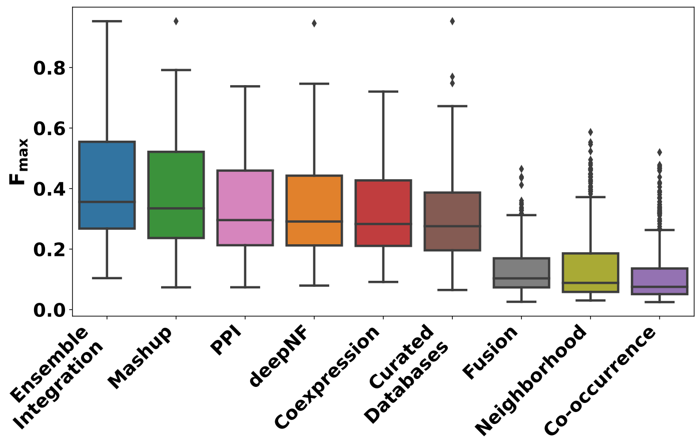

(c) GO terms with 200 to 500 annotations (FDR of EI vs deepNF $< 2 \times 10^{-16}$, EI vs Mashup $= 3.52 \times 10^{-10}$, EI vs all individual modalities: $< 2 \times 10^{-16}$).

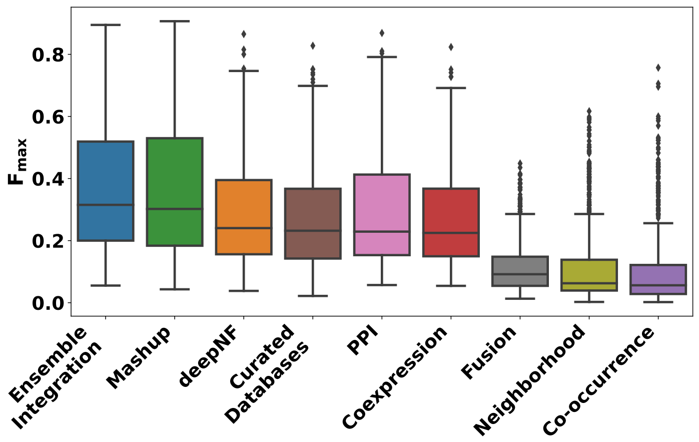

(d) GO terms with 100 to 200 annotations (FDR of EI vs deepNF $< 2 \times 10^{-16}$, EI vs Mashup $= 0.001$, EI vs all individual modalities $< 2 \times 10^{-16}$).

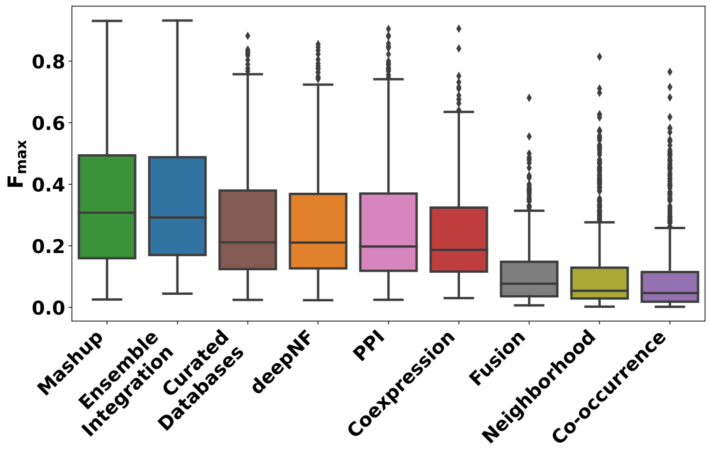

(e) GO terms with 50 to 100 annotations (FDR of EI vs deepNF $< 2 \times 10^{-16}$, EI vs Mashup $= 7.77 \times 10^{-4}$, EI vs all individual modalities $< 2 \times 10^{-16}$).

Supplementary Fig. 3: **Distributions of performances of the protein function prediction approaches tested in this work across GO terms grouped by the number of human genes annotated to them.** Performance was measured in terms of the F_{max} score. Also shown are the FDR values representing the statistical significance of the comparative performance of EI vs deepNF, Mashup and individual STRING data modalities.

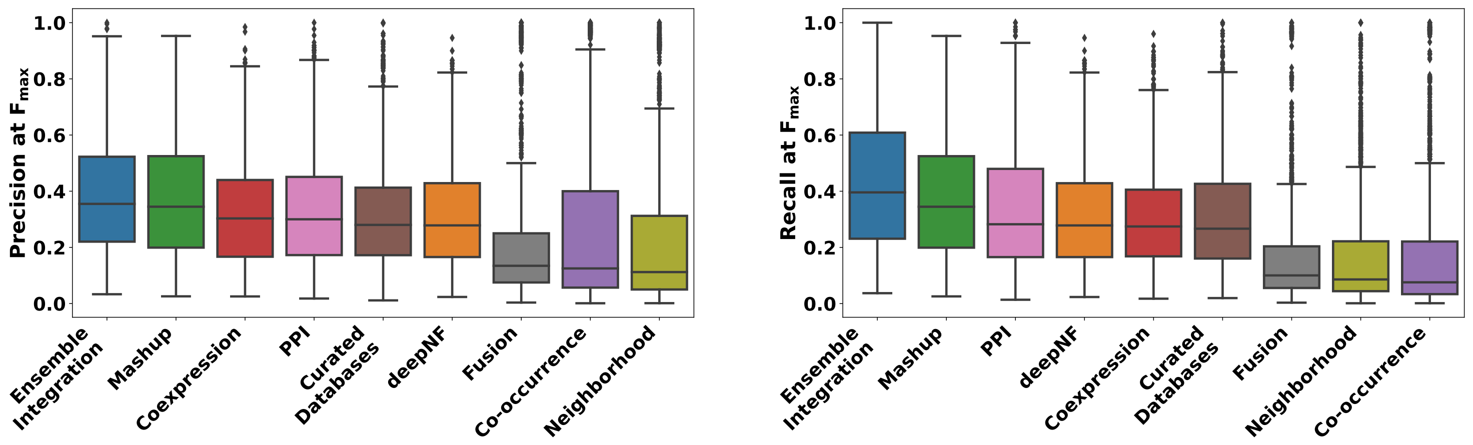

(a) Distribution of precision at F_{max} across all the GO terms tested (FDR of EI vs deepNF $< 2 \times 10^{-16}$, EI vs Mashup $= 0.006$, EI vs all individual modalities $< 2 \times 10^{-16}$).

(b) Distribution of recall at F_{max} across all the GO terms tested (FDR of EI vs deepNF $< 2 \times 10^{-16}$, EI vs Mashup $< 2 \times 10^{-16}$, EI vs all individual modalities $< 2 \times 10^{-16}$).

Supplementary Fig. 4: **Distributions of (a) precision and (b) recall yielding the F_{max} values reported in Fig. 3 for all the protein function prediction approaches, data modalities and GO terms tested in this work.** Also shown are the FDR values representing the statistical significance of the comparative performance of EI vs deepNF, Mashup and individual STRING data modalities.

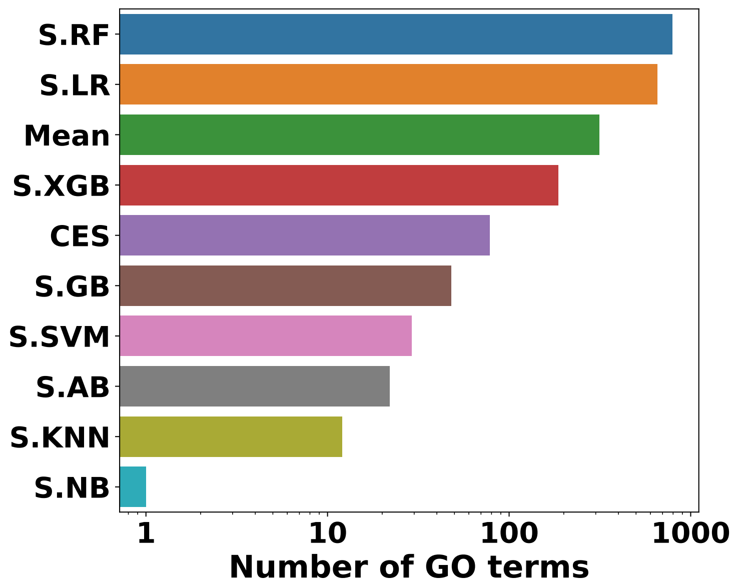

Supplementary Fig. 5: **Distribution of best-performing heterogeneous ensemble methods used within EI for protein function prediction.** This distribution was calculated across all the GO terms and the eleven ensemble methods tested. The Y-axis shows the names of the ensemble methods. The names with prefix 'S.' denote stacking with the classification algorithm named in the suffix, e.g., 'S.RF' stands for stacking with random forest. The X-axis shows the count (in logarithmic scale) of the GO terms for which each ensemble method showed the best performance. Note that stacking with decision tree (S.DT) is not shown here, since it was not found to be the best performer for any term, i.e., it's count on the X-axis was zero.

Supplementary Tables

Table 1: Details of the clinical variables in electronic health records (EHRs), organized by the modalities they belonged to, that were used to predict mortality due to COVID-19 (Section 2.3.2 of the main text). Also provided are the units of the laboratory tests, as well as the exact or prefix of ICM-10-CM diagnosis code used for determining the values of the features in the co-morbidities modality.

Admission (variables collected at the beginning of a patient's hospital encounter)

**Feature**	Description (units where applicable)
Age	The patient's age rounded down to nearest integer at the time of their hospital encounter.
Diastolic BP	The patient's first diastolic blood pressure reading taken during the encounter (mmHg).
Heart Rate	The patient's first recorded heart rate during the encounter (beats/minute).
Oxygen Saturation	The patient's first recorded oxygen saturation during the encounter (percentage).
Respiratory Rate	The patient's first recorded respiratory rate during the encounter (breaths/minute).
Systolic BP	The patient's first systolic blood pressure reading taken during the encounter (mmHg).
Temperature	The patient's first recorded temperature during the encounter (Fahrenheit).
Race/Ethnicity - Amercian Indian or Alaska Native	Binary variable indicating if the patient identified as Amercian Indian or Alaska Native.
Race/Ethnicity - Asian	Binary variable indicating if the patient identified as Asian.
Race/Ethnicity - Black or African-American	Binary variable indicating if the patient identified as Black or African-American.
Race/Ethnicity - White	Binary variable indicating if the patient identified as White.
Race/Ethnicity - Hispanic	Binary variable indicating if the patient identified as Hispanic.
Race/Ethnicity - Unknown	Binary variable indicating if the patient identified as unknown Race/Ethnicity.
Race/Ethnicity - Other	Binary variable indicating if the patient identified as other Race/Ethnicity.
Sex - Female	Binary variable indicating if the patient identified as female.
Sex - Male	Binary variable indicating if the patient identified as male.
Smoking Status - Never	Binary variable indicating if the patient never smoke.
Smoking Status - Not Asked	Binary variable indicating if the patient was not asked about their smoking status.
Smoking Status - Passive	Binary variable indicating if the patient's smoking status is passive.
Smoking Status - Quit	Binary variable indicating if the patient quit smoking.
Smoking Status - Yes	Binary variable indicating if the patient is still smoking.

Co-morbidities (binary variables indicating various morbidities diagnosed by ICD-10-CM codes)

**Feature**	**Description**
Acute Kidney Injury	Diagnosed by an ICD-10-CM code beginning with N17.
Acute Myocardial Infarction	Diagnosed by an ICD-10-CM code beginning with I21.
Acute Venous Thromboembolism	Diagnosed by an ICD-10-CM code beginning with I26 or I82.4.
Alcoholic/Non-alcoholic Liver Disease	Diagnosed by ICD-10-CM codes K75.81 or K76.0.

(continued on the next page)

Table 1: (continued from the previous page)

Acute Respiratory Distress Syndrome	Diagnosed by ICD-10-CM code J80.
Asthma	Diagnosed by an ICD-10-CM code beginning with J45.
Atrial Fibrillation	Diagnosed by an ICD-10-CM code beginning with I48.
Cancer Flag	Diagnosed by an ICD-10-CM code beginning with C.
Cerebral Infarction	Diagnosed by an ICD-10-CM code beginning with I63.
Chronic Kidney Disease	Diagnosed by ICD-10-CM codes E08.22, E09.22, E10.22, E11.22, E13.22 or beginning with I12, I13, N18.
Chronic Viral Hepatitis	Diagnosed by an ICD-10-CM code beginning with B18.
Chronic Obstructive Pulmonary Disease	Diagnosed by ICD-10-CM codes beginning with J41, J43 or J44.
Coronary Artery Disease	Diagnosed by ICD-10-CM codes beginning with I21, I22, I23, I24 or I25.
Crohns Disease	Diagnosed by ICD-10-CM codes beginning with K50.
Diabetes	Diagnosed by ICD-10-CM codes beginning with E08, E09, E10, E11, E13, O24.0, O24.1, O24.3 or O24.8.
Heart Failure	Diagnosed by ICD-10-CM codes beginning with I50.
HIV Flag	Diagnosed by ICD-10-CM codes B20, B97.35, O98.7, O98.71, O98.711, O98.712, O98.713, O98.719, O98.72, O98.73 or Z21.
Hypertension	Diagnosed by ICD-10-CM code I10.
Intracerebral Hemorhage	Diagnosed by an ICD-10-CM code beginning with I61.
Obesity	Diagnosed by ICD-10-CM code E66.1, E66.2, E66.8 or E66.9.
Obstructive Sleep Apnea	Diagnosed by ICD-10-CM code G47.33.
Ulcerative Colitis	Diagnosed by an ICD-10-CM code beginning with K51.

Laboratory Tests (continuous variables measured from a patient's blood sample, unless a different sample source is specified)

**Feature**	**Description**	**Unit**
Albumin	Amount of albumin	gram/deciliter
Alanine Transaminase	Amount of alanine transaminase.	unit/liter
Anion Gap	A measure of acid-base balance	milliequivalent/liter
Aspartate Aminotransferase	Amount of aspartate aminotransferase	unit/liter
Basophil (Count)	Count of basophil in white blood cell	($10^{-3}\times$ count)/microliter
Basophil (Percentage)	Percentage of basophil in white blood cell	percentage
Blood Urea Nitrogen	Amount of nitrogen in the waste product urea	milligram/deciliter.
C-reactive Protein	Amount of C-reactive protein	milligram/liter
Calcium	Amount of calcium	milligram/deciliter
Chloride	Amount of chloride	milliequivalent/liter
CO_2 Total	Amount of carbon dioxide	milliequivalent/liter
D-dimer	Fibrinogen equivalent units of d-dimer	microgram/milliliter

(continued on the next page)

Table 1: (continued from the previous page)

Estimated Glomerular Filtration Rate	The estimated glomerular filtration rate	milligram/minute/ ($1.73\times$meter-squared)
Eosinophil (Count)	Count of eosinophils in white blood cells	($10^{-3}\times$ count)/microliter
Eosinophil (Percentage)	Percentage of eosinophils in white blood cells	percentage
Ferritin	Amount of ferritin	nanogram/microliter
Glucose	Amount of glucose	milligram/deciliter
Venous HCO_3	Amount of bicarbonate in venous blood	milliequivalent/liter
Hemoglobin	Amount of hemoglobin	gram/deciliter
Lactate Dehydrogenase	Amount of lactate dehydrogenase	unit/liter
Lymphocyte (Count)	Count of lymphocytes in white blood cells	($10^{-3}\times$ count)/microliter
Lymphocyte (Percentage)	Percentage of lymphocytes in white blood cells	percentage
Mean Corpuscular Hemoglobin	Average amount of corpuscular hemoglobin	gram/deciliter
Mean Corpuscular Hemoglobin Concentration	The average concentration of corpuscular hemoglobin	gram/deciliter
Mean Corpuscular volume	Average corpuscular volume	femtolitre
Mean Platelet Volume	Average platelet volume	femtolitre
Monocyte (Count)	Count of monocytes in white blood cells	($10^{-3}\times$ count)/microliter
Monocyte (Percentage)	Percentage of monocytes in white blood cells	percentage
Neutrophil (Count)	Count of neutrophils in white blood cells	($10^{-3}\times$ count)/microliter
Neutrophil (Percentage)	Percentage of neutrophils in white blood cells	percentage
Venous O_2 Saturation	Oxygen saturation in venous blood	percentage
Venous PCO_2	The partial pressure of carbon dioxide in venous blood	mmHg
Venous pH	The pH of venous blood	No unit
Platelet	Amount of platelets	($10^{-3}\times$ count)/microliter
Venous PO_2	The partial pressure of oxygen in venous blood	mmHg
Potassium	Amount of potassium	millimoles/liter
Procalcitionin	Amount of procalcitionin	nanogram/milliliter
RBC Count	Red blood cell count	($10^{-6}\times$ count)/microliter
Serum Creatinine	Amount of creatinine	milligram/deciliter
Sodium	Amount of sodium	millimoles/liter
Total Bilirubin	Total amount of bilirubin	milligram/deciliter
Total Protein	Total amount of two classes of proteins (albumin and globulin)	gram/deciliter
Troponin I	Amount of troponin I	nanogram/milliliter
WBC	Amount of white blood cells	($10^{3}\times$ count)/milliliter
**Vital Signs (maximum and/or minimum of continuous-valued measurements during a patient's hospital encounter)**		
**Feature**	**Description**	Unit
Maximum Diastolic BP	Maximum diastolic blood pressure	mmHg
Minimum Diastolic BP	Minimum diastolic blood pressure	mmHg

(continued on the next page)

Table 1: (continued from the previous page)

Maximum Heart Rate	Maximum heart rate	Beats/minute
Minimum Heart Rate	Minimum heart rate	Beats/minute
Minimum O_2 Saturation	Minimum oxygen saturation	Percentage
Maximum Respiratory Rate	Maximum respiratory rate	Breaths/minute
Maximum Systolic BP	Maximum systolic blood pressure	mmHg
Minimum Systolic BP	Minimum systolic blood pressure	mmHg
Maximum Temperature	Maximum temperature	Fahrenheit

Table 2: Ten highest contribution features for predicting mortality due to COVID-19 identified using the XGBoost method in Vaid *et al.* (2020)'s study (details of the features are in Supplementary Table 1).

**Modality**	**Feature**
Admission	Age
Laboratory Tests	Troponin I
Laboratory Tests	Platelet
Vital Signs	Minimum O_2 Saturation
Laboratory Tests	C-Reactive Protein
Laboratory Tests	Aspartate Aminotransferase
Laboratory Tests	Glucose
Laboratory Tests	Calcium
Laboratory Tests	Blood Urea Nitrogen
Laboratory Tests	Procalcitonin

References

Radivojac, P. *et al.* (2013). A large-scale evaluation of computational protein function prediction. *Nature Methods*, **10**(3), 221–227.

Vaid, A. *et al.* (2020). Machine learning to predict mortality and critical events in a cohort of patients with covid-19 in new york city: Model development and validation. *J Med Internet Res*, **22**(11), e24018.

Walsh, I. *et al.* (2021). DOME: recommendations for supervised machine learning validation in biology. *Nature methods*, **18**(10), 1122–1127.

Whalen, S. *et al.* (2016). Predicting protein function and other biomedical characteristics with heterogeneous ensembles. *Methods*, **93**, 92–102.
